# Impulse control disorders in Parkinson’s disease: A systematic review on the psychometric properties of the existing measures

**DOI:** 10.1371/journal.pone.0217700

**Published:** 2019-06-04

**Authors:** Viola Angela Izzo, Maria Anna Donati, Silvia Ramat, Caterina Primi

**Affiliations:** 1 NEUROFARBA Department, Section of Psychology, University of Florence, Italy; 2 Department of Developmental and Social Psychology, Sapienza University of Rome, Rome, Italy; 3 Neurologia 1—AOUC Azienda Ospedaliero-Universitaria Careggi, Florence, Italy; Philadelphia VA Medical Center, UNITED STATES

## Abstract

**Background:**

A significant percentage of patients suffering from Parkinson’s Disease (PD) experience Impulse Control Disorders (ICDs), contributing to reduced quality of life. As they can be managed by reducing the dopamine dosage, the detection of their presence is crucial for PD treatment plan. Nevertheless, they tend to be under-recognized in clinical practice, since routine screening is not common–despite existing instruments that may support clinicians. This work presents a systematic review on the psychometric properties of instruments measuring ICDs in PD, to test whether clinicians dispose of valid tools that may help them in clinical assessment.

**Method:**

A systematic literature search in three databases (EMBASE, MEDLINE, and PsycINFO) was conducted. Quality of the instruments’ psychometric properties was evaluated with Terwee et al.’s criteria, and methodological quality of the studies was evaluated with the COSMIN Checklist.

**Results:**

Ten studies examining seven instruments were selected. The *Questionnaire for Impulsive-Compulsive Disorders in Parkinson’s Disease* (QUIP) and the *Ardouin Scale of Behavior in Parkinson’s Disease* (ASBPD) resulted to be the best from a psychometric point of view.

**Conclusions:**

Though the gold standard for diagnosis remains a detailed diagnostic interview, this review will encourage clinicians to use validated tools to accurately assess ICDs.

## Introduction

Parkinson’s disease (PD) represents the second most common neurodegenerative disorder after Alzheimer’s disease, with a prevalence of 1–2% in those over the age of sixty and 3–5% in those over 85 years old [1, 2]. Symptoms mainly involve movement, so that PD’s most well-known signs are tremor, bradykinesia, muscle stiffness and impaired balance and coordination. Nonetheless, there is well-established evidence that cognition and emotion are also impaired, with psychiatric symptoms being present in more than 60% of PD patients [3]. Among the non-motor symptoms, concern has arisen for a peculiar group of impulsive behaviors, known as Impulse Control Disorders (ICDs), which occur in up to 20% of PD patients [4]. ICDs are a group of behaviors characterized by a failure to resist an impulse, drive, or temptation to perform an act that is harmful to oneself or to others [5]. They include pathological gambling [6], hypersexuality [7], compulsive eating [8], and compulsive buying [9]. Along with them, other related impulsive-compulsive behaviors occur, including punding [10], hobbyism [11], walkabouts [12], and a peculiar condition named Dopamine Dysregulation Syndrome (DDS), defined as a compulsive use of dopamine medications despite adequate motor benefits and the annoying consequences [13].

Although evidence of ICDs in PD patients has been reported even before [14], research on this topic flourished in the 2000s, when many studies found that PD patients reported those impulsive behaviors though they had never experienced them prior to the disease [15–18]. The first case studies led to the hypothesis that the onset of those peculiar behaviors may be due to the pharmacological dopaminergic therapy used to treat PD, particularly of dopamine agonists [19, 20]. Evidence of the link between ICDs and dopaminergic therapy is provided by distinct aspects: 1) prevalence rates of ICDs seem to be higher in patients with PD rather than in the general population; for example, lifetime prevalence rates are between 3% and 8% for pathological gambling in PD versus a 0.3% to 2% rate in the latter [20–22]; 2) prevalence rates are even higher in PD patients using dopamine agonists, with an estimate of 13.7% [23]; 3) rates of pathological gambling are high also in other medical conditions that require the use of dopamine agonists [24, 25]. Referring to the onset time of ICDs, though almost all studies examining ICDs in PD have been cross-sectional, a recent prospective study found that 39% of patients developed an ICD within four years from the initiation of the dopaminergic treatment, though they had never experienced them before [26].

One possible explanation for the association between ICDs and dopaminergic therapy in PD patients is the “overstimulation hypothesis” in the ventral striatum [22, 27], that is ICDs may result from an excessive dopaminergic stimulation of those areas involved in the reward system. While dopaminergic loss in PD affects above all the dorsal striatum, responsible for movements, the ventral striatum, which regulates reward responsiveness, remains relatively intact, especially in the early stages. As such, though providing substantial benefits to motor symptoms, dopaminergic treatment may increase reward sensitivity and promote the onset of the ICDs [27, 28].

As previously mentioned, dopamine agonists seem to be strongly associated with the development of ICDs–though a lesser association was also found with high doses of levodopa [4]. This may be because, compared to levodopa, most dopamine agonists have a preferential selectivity for D3 and D2 receptors rather than D1 type [29]: while D1 receptors are mostly concentrated in dorsal striatum, where they facilitate motor function, D2-like receptors are set in the limbic areas, including ventral striatum, where they play an important role in reward and addiction circuits. Hence, dopamine agonists may activate limbic mechanisms more than motor ones.

However, dopaminergic treatment cannot be considered as the only factor responsible for ICDs in PD. In fact, though a considerable percentage of PD patients experiences at least one ICD, they represent a minority of the PD patients that are treated with dopaminergic medication [27], so other responsible factors have been proposed [28, 30]. Some of them are related to PD, such as an earlier onset of the disease, whereas some others refer to personal factors, including a familiar history of addictions, male gender, and, above all, personality traits. Among them, impulsivity is one of the main suspects [30–32].

PD patients suffering from ICDs have greater functional impairment in activities of daily living compared with PD controls [4, 19]. In fact, ICDs usually predict reduced quality of life, mainly in terms of emotional well-being, with the presence of depressed mood and/or irritability [33, 34]. They are also associated with increased social impairment, including divorce, bankruptcy, incarceration, and attempted suicide [35, 36]. Nevertheless, ICDs in PD patients tend to be under-recognized and under-managed in clinical practice [20, 33, 37]. Under-recognition may be attributed both to the fact that patients may under-report symptoms, and that routine screening is not common [4]. In fact, precisely screening for impulsive behaviors using psychometrically-based tools does not usually represent a standard procedure, since, in clinical practice physicians often ask about ICDs but do not commonly use more comprehensive screening tools. Yet, case reports suggest that ICDs often resolve after reducing the dose of the existing DA, even when compensating with an increase in levodopa dosage [38, 39]. Thus, testing the presence of those impulsive behaviors may be crucial for making adjustments to the treatment plan.

As such, the use of psychometrically-based instruments aiming to assess the presence of these aspects–and, if present, their frequency and severity–is highly recommended during the diagnostic process and the clinical evaluation, as to develop a case-by-case strategy of intervention. In support of the increasing interest towards the instruments assessing ICDs, a recent semi-systematic review reported a non-exhaustive list of some existing tools and described their main features, though it did not report any information about their psychometric properties [40]. Nonetheless, not all the existing instruments aiming to assess the same clinical feature are equally good in terms of psychometric properties; that is, there may be differences in terms of their ability to effectively and precisely measure the considered aspect. As so, to be aware of the quality of the tool before administering it, the analysis of its measurement properties would be needed.

Given the huge interest in assessing ICDs in PD patients, the aim of the present work was to systematically review the psychometric properties of the instruments used in research and practice in patients with PD. In detail, the specific aims of this study were fourfold: 1) to provide a list of the instruments assessing multiple ICDs, whose psychometric properties have been investigated in PD patients–starting with the consideration that routine screening of ICDs in PD patients is not common [4], aiming to practically help clinicians in easily, rapidly evaluating the presence of these behaviors, we decided to focus only on those instruments that assessed more than one ICD at the same time; 2) to provide information on their specific psychometric characteristics; 3) to qualitatively assess the detected instruments in light of their psychometric properties; and 4) to assess the methodological quality of the included studies. In sum, this systematic review aimed to increase knowledge about the psychometric properties of instruments assessing ICDs in PD, thus identifying potential areas for improvement and further developments in this field from a psychometric perspective.

## Materials and methods

Methods of this systematic review were specified before starting the study according to the Preferred Reporting Items for Systematic Reviews and Meta-Analyses (PRISMA) Guidelines [41]. The methods are specified as follows.

### Search criteria

A comprehensive literature search was conducted on electronic databases including EMBASE, MEDLINE, and PsycINFO from 2000, when the first cases of PD patients experiencing ICDs were reported, to June 2018. The following research equation was used for Embase: ‘‘Parkinson*”[Title/Abstract/Author Keywords] AND (‘‘Impuls*” [Title/Abstract/Author Keywords]). The keyword “Impuls*” was chosen because ICD is the acronym for Impulse Control Disorder, so using Impuls* all records dealing with ICDs would have resulted from research. For MEDLINE the research equation was: (Parkinson*[Title/Abstract]) AND Impuls*[Title/Abstract]. Eventually, for PsychInfo the equation was: Any Field: Parkinson* *AND* Any Field: Impuls*. An additional article [42] was considered for the screening procedure after reading its references in articles resulting from databases.

### Selection criteria

The eligibility criteria for the selection of the publications to be reviewed were articles that: (1) were limited to humans; (2) were conducted on populations affected by PD; (3) reported the psychometric evaluation of a tool assessing multiple ICDs–which led to excluding some screening tools such as the Minnesota Impulsive Disorders Interview [43], which, though being frequently used with PD patients, has never been validated in this population; (4) were peer-reviewed; and (5) were published in English. Studies were excluded if (1) they were meeting abstracts or materials published in the form of thesis, book chapters, and manuals; and (2) they were systematic reviews; and (3) full-text of the eligible article was not provided.

### Search procedures

All search outputs were independently examined by the first and second authors to determine eligibility for inclusion. When disagreement occurred, the third author was consulted until a consensus was reached. Using the search keywords, the titles and abstracts were first screened to identify eligible articles. Full texts were obtained for those abstracts that were rated positive to enable further evaluation, according to which articles were included or not.

### Quality assessment

Regarding the investigation of psychometric properties of the identified instruments, several operationally defined indicators were examined according to the Terwee et al. criteria [44] and to the modifications proposed by Park, Reilly-Spong, and Gross [45]. The first domain was *reliability*, defined as the degree to which the measurement is free from measurement error. Specifically, it was analyzed in terms of three measurement properties: 1) internal consistency, which is the extent to which items in a scale are correlated (i.e., homogeneous); 2) reliability–divided into a) test-retest reliability, which is the extent to which the test for patients who have not changed produce the same results in different occasions over time; and b) inter-rater reliability, which is the extent to which scores for patients who have not changed are the same when evaluated by different raters; and 3) measurement error, which is the systematic and random error that is not attributed to true changes in the underlying construct, and it is adequate if the smallest detectable change (SDC) on the instrument is less than the minimal important change (MIC) [46].

The second domain was *validity*, which was examined to test whether the instruments actually measured the construct(s) they purport to measure. Different measurement properties of validity were assessed, including 1) content validity, in particular in the form of face validity, which is the degree to which the items of an instrument seem to be an adequate reflection of the construct to be measured; 2) construct validity, whose examined aspects were a) structure validity, defined as the analysis of dimensionality of the construct, the degree to which the scores of an instrument are an adequate reflection of the dimensionality of the construct to be measured; and b) hypotheses-testing, defined as the degree to which the scores of an instrument are consistent with hypotheses, e.g., with regards to relationships to scores of other instruments or differences between relevant groups; and 3) criterion validity, which is the degree to which the scores of an instrument are an adequate reflection of a ‘‘gold standard”.

Moreover, *responsiveness*, defined as the ability of an instrument to detect changes over time in the construct to be measured, was evaluated. Eventually, *interpretability*, which is the degree to which one can assign qualitative meaning to quantitative scores, was examined.

Based on Terwee et al.’s criteria [44] with the revisions of Park et al. [45], the psychometric properties of the selected articles were rated as “positive”, “negative”, or “indeterminate”. Ratings equal to “0” were given when no information was available (Table 1).

**Table 1:** Relevant psychometric properties and each criterion used and relative definition adapted from Terwee et al. [44] and Park et al. [45] criteria for measurement properties.

Domain	Measurement Property	Aspects of measurement property	Rating	Quality Criteria
**Reliability**	Internal consistency		+	(Sub)scale unidimensional AND Cronbach’s alpha(s) ≥.70
			?	Dimensionality not known OR Cronbach’s alpha not determined
			-	(Sub)scale not unidimensional OR Cronbach’s alpha(s) < .70
	Reliability		+	ICC/weighted Kappa ≥ .70 OR Pearson’s r ≥ .80
			?	Neither ICC/weighted Kappa, nor Pearson’s r determined
			-	ICC/weighted Kappa < .70 OR Pearson’s r < .80
	Measurement error		+	MIC > SDC OR MIC outside the LOA
			?	MIC not defined
			−	MIC ≤ SDC OR MIC equals or inside LOA
**Validity**	Content validity	Face validity	+	The target population considers all items in the questionnaire to be relevant AND considers the questionnaire to be complete
			?	No target population involvement OR no assessment of completeness or comprehensiveness
			-	The target population considers items in the questionnaire to be irrelevant OR considers the questionnaire to be incomplete
	Construct validity	Structural validity	+	Factors should explain at least 50% of the variance OR good or adequate fit by goodness-of-fit criteria for a CFA or EFA^a^
			?	Explained variance not mentioned OR equivocal fit by goodness-of-fit criteria for a CFA or EFA^a^
			-	Factors explain < 50% of the variance OR poor fit by goodness-of-fit criteria for a CFA or EFA^a^
		Hypotheses-testing	+	At least 75% of the results are in accordance with the hypotheses AND correlation with related constructs is higher than with unrelated constructs OR no evidence of DIF
			?	Solely correlations determined with unrelated constructs OR ≥ 50% but < 75% of the results are in accordance with the hypotheses OR possible DIF
			-	Less than 50% of the results are in accordance with the hypotheses OR correlation with related constructs is lower than with unrelated constructs OR notable evidence of DIF
	Criterion validity		+	Convincing arguments that gold standard is ‘‘gold” AND correlation with gold standard >.70
			?	No convincing arguments that gold standard is ‘‘gold” OR doubtful design or method
			-	Correlation with gold standard < .70, despite adequate design and method
**Responsiveness**			+	Correlation of changes with an instrument measuring change in the same construct ≥ .50 OR at least 75% of the results are in accordance with the hypotheses OR AUC ≥ .70 OR correlation of changes with related constructs is higher than with unrelated constructs OR statistically significant paired t test
			?	Solely correlations determined with unrelated constructs
			-	Correlation of changes with an instrument measuring change in the same construct < .50 OR < 75% of the results are in accordance with the hypotheses OR AUC < .70 OR correlation of changes with related constructs is lower than with unrelated constructs OR not significant paired t test
**Interpretability**			+	Mean and SD scores presented of at least four relevant subgroups of patients and MIC defined
			?	Doubtful design or method OR less than four subgroups OR no MIC defined
			-	No information found on interpretation

*Note*: MIC: minimal important change, SDC: smallest detectable change, LOA: limits of agreement, ICC: Intraclass correlation coefficient, DIF: Differential item functioning, AUC: Area under the curve, SD: standard deviation. + positive rating, ? indeterminate rating,—negative rating.

^a^Good or Adequate fit: comparative fit index (CFI) ≥ .90, root mean square error of approximation (RMSEA) ≤ .08, standardized root mean square residual (SRMR) < .10; Inadequate fit: CFI ≤ .85, RMSEA ≥ .10, SRMR ≥ .10; Indeterminate fit: the values of fit indexes ranged in between the adequate criteria and inadequate criteria [45].

As a combination of the list proposed by Terwee et al. [44] and the COSMIN checklist (Consensus-based Standards for the selection of health status Measurement Instruments) [47, 48] has been recommended in assessing the quality of questionnaires [47], besides the qualitative analysis of the psychometric properties, the methodological quality of the studies on the measurement properties was examined according to the COSMIN checklist. The COSMIN contains four steps and 12 boxes. Ten of them aim to assess whether a study meets the standards for good methodological quality for each psychometric property separately (Boxes A to J), whereas two boxes examine general requirements for articles involving Item Response Theory (IRT) methods and general requirements for the generalizability of the results. Each box contains 4-to 18 items, with 119 items in total; each item is rated on a 4-point rating scale, equivalent to excellent (+++) when there is evidence that the methodological quality of that peculiar aspect is adequate, good (++) when relevant information is not reported in the article but it can be assumed that the quality aspect is adequate, fair (+) when it is doubtful whether the methodological quality aspect is adequate, and poor (0) when there is evidence that the methodological quality aspect is not adequate. The overall score *per* box is determined by the item with the lowest score [47, 48].

## Results

**Fig 1:**
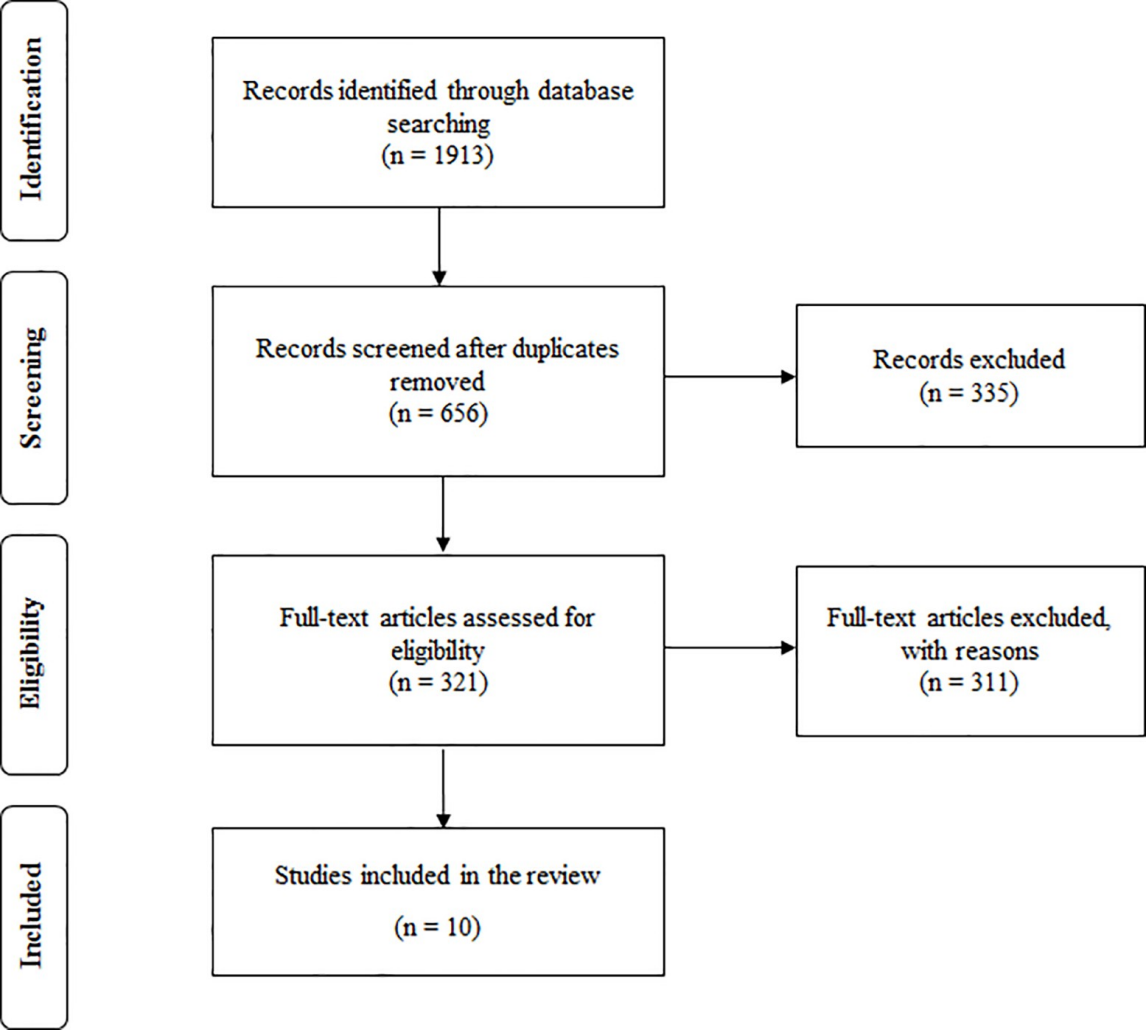
Flowchart of the search strategy and selection of articles.

The search returned 1913 publications. After excluding 1257 duplicates, we reviewed the titles and abstracts for each of the 656 remaining publications. Among them, 321 studies met the inclusion criteria of being peer-reviewed full-text articles published in English that examined ICDs in PD patients. However, of those 321 articles, 311 were removed as they administered tools assessing ICDs in PD patients, but did not examine their psychometric properties. As a final result, ten studies were identified (Fig 1).

Those ten eligible articles reported the psychometric properties of seven instruments, although three of them were represented by the *Questionnaire for Impulsive-Compulsive Disorders in Parkinson’s Disease* (QUIP) [49], and two modified versions–i.e., the *Questionnaire for Impulsive-Compulsive Disorders in Parkinson’s Disease-Short version* (QUIP-S) [49], and the *Questionnaire for Impulsive-Compulsive Disorders in Parkinson’s Disease-Rating Scale*, which includes both the frequency and the severity of symptoms belonging to the ICDs sphere (QUIP-RS) [50]. The other tools were: The *Dopamine Dysregulation Syndrome-Patient and Caregiver Inventory* (DDS-PC) [51], the *Ardouin Scale of Behavior in Parkinson’s Disease* (ASBPD, developed by Ardouin et al. [52], and validated by Rieu et al. [42]), the *Impulse Control Disorders and Related Conditions Questionnaire* (ICDRC) [53], and the *Parkinson’s Impulse Control Scale for the severity rating of Impulse-Control Behaviors* (PICS) [54]. The selected tools are reported in Table 2, along with a brief description of their characteristics.

**Table 2:** Descriptive characteristics of the selected articles.

Instrument (Authors)	Content validity (conceptualization of the construct)	Main test characteristics (i.e., number of items, factors, response mode, administration time)	Studies examining the psychometric properties in PD patients	Sample involved	Country
***Ardouin Scale of Behavior in Parkinson’s Disease* (ASBPD) [52]**	General Psychological state, including Impulse Control Disorders	Semi-structured interview with 21 items assessing general psychological state (including depressive mood, hypomanic or manic mood, anxiety, irritability, hyper-emotivity, psychotic symptoms), apathy, nonmotor fluctuations, and ICDs and related behavior symptoms (nocturnal hyperactivity, diurnal somnolence, excessive eating behavior, creativity, hobbyism, punding, risk-seeking behavior, compulsive shopping, pathological gambling, hypersexuality, and compulsive dopaminergic medication use). Each item rates the frequency and intensity of symptoms’ occurrence in the preceding month on a 0-to 4 ranging scale, with 0 indicating no modification of the patient’s usual habits, and a score >2 reflecting marked to severe modification and the presence of a maladaptive pathological behavior.	Rieu et al. [42]	260 PD patients from France, Spain, the UK and the USA; at least 60 patients were included for each language group	France, Spain, UK, and USA
***Dopamine Dysregulation Syndrome-Patient and Caregiver Inventory* (DDS-PC) [51]**	Impulse Control Disorders	Self-report questionnaire. It includes 45 items on a five-point Likert scale referring to a 12-month period, examining compulsive medication use, pathological gambling, hypersexuality, binge eating, punding, compulsive shopping, reckless driving, and violent behaviors. There are two parallel forms: one for patients, and one for their caregivers.	Cabrini et al. [51]	38 PD patients and their caregivers	Italy
***Impulse Control Disorders and Related Conditions Questionnaire* (ICDRC) [53]**	Impulse Control Disorders	Self-report questionnaire. It consists of 12 questions on a five-point Likert scale from 0 (= never) to 4 (= always) evaluating the frequency and the consequences of pathological gambling, compulsive shopping, compulsive eating, hypersexuality, punding, and dopamine dysregulation syndrome. There are two parallel forms: one for patients, and one for their caregivers.	Baumann-Vogel et al. [53]	78 PD patients and 64 caregivers	Switzerland
***Parkinson’s Impulse Control Scale for the severity rating of Impulse-Control Behaviors* (PICS) [54]**	Impulse Control Disorders	Semi-structured interview. It measures both the intensity of each ICD (i.e., gambling, shopping, eating, hypersexuality, punding, hobbyism, and compulsive overuse of medication) and the individual and social impact within a timeframe of 1 month. Each ICD subscale includes a 3-item screening section with yes/no responses, which, if positive, are followed by further questions evaluating symptoms’ intensity and impact.	Okai et al. [54]	92 PD patients, divided into 45 PD patients with ICDs and 41 without ICDs	United Kingdom
***Questionnaire for Impulsive-Compulsive Disorders in Parkinson’s Disease* (QUIP) [49]**	Impulse Control Disorders	Self- or rater-administered questionnaire. It consists of three sections: (1) ICDs: gambling, sexual, buying and eating behaviors (there are five questions per each ICD); (2) other compulsive behaviors: punding, hobbyism and walkabout (there are three distinct introductory questions and two common additional questions for the three behaviors); and (3) compulsive PD medication use (it includes five questions). Answers are organized into a dichotomous choice (yes or no) investigating behaviors lasting at least 4 weeks that occurred anytime after PD onset. Administration time is approximately 5 minutes.	Weintraub et al. [49]	• 157 patients with idiopathic PD	USA
			Papay et al. [55]	• 71 patients with idiopathic PD and their caregiver	USA
			Tanaka et al. [56]	• 118 PD patients	Japan
			Probst et al. [57]	• 150 PD patients	Germany
***Questionnaire for Impulsive-Compulsive Disorders in Parkinson’s Disease-Short version* (QUIP-S) [49]**	Impulse Control Disorders	Self- or rater-administered questionnaire. It consists of 13 items covering the three sections of the full-length form (two questions for each of the four ICDs, three questions for other compulsive behaviors, and two questions for compulsive medication).	Weintraub et al. [49]	• 157 patients with idiopathic PD	USA
			Krieger et al. [58]	• 30 patients with idiopathic PD	Brazil and other Portuguese-speaking countries
***Questionnaire for Impulsive-Compulsive Disorders in Parkinson’s Disease-Rating Scale* (QUIP-RS) [50]**	Impulse Control Disorders	Self- or rater-administered questionnaire. It measures the frequency as well as the severity of symptoms. It consists of 4 primary questions (pertaining to commonly reported thoughts, urges/desires, and behaviors associated with ICDs), each applied to the 4 ICDs (compulsive gambling, buying, eating, and sexual behavior) and 3 related disorders (medication use, punding, and hobbyism). It uses a 5-point Likert scale (score 0–4 for each question) to detect the frequency of behaviors. Patients are required to answer questions based on behaviors that occurred in the preceding 4 weeks.	Weintraub et al. [50]	• 104 patients with idiopathic PD • Subsets of patients were enrolled to examine interrater reliability (n = 104), retest reliability (n = 63), and responsiveness to change (n = 29)	USA
			Probst et al. [57]	• 144 PD patients	Germany

### Overview of included instruments

Five of the tools examined in the selected articles are self-report questionnaires (though the three versions of the QUIP can be both self- or rater-administered), whereas two measures are semi-structured interviews (i.e., the ASBPD and the PICS). While the other instruments specifically aim to examine ICDs and related behaviors–in detail: gambling, sexual, buying, and eating behaviors, punding, hobbyism, and compulsive medication use–the ASBPD is a wider measure assessing general psychological state, including depressive and manic mood, anxiety, psychotic symptoms, and ICDs in PD patients.

Concerning the structure of the instruments, the number of total items is limited, with a maximum of 45 items. This could be due to the consideration of the characteristics of the PD population, which, though not reporting a significant cognitive impairment, as a clinical sample, does have to face some difficulties when completing lengthy measures.

Regarding the purpose of the selected instruments, some of them are screening tools (i.e., the QUIP and the QUIP-S). Others are rating scales, which can determine the frequency and/or severity of ICDs (i.e., the DDS-PC, the QUIP-RS, the ASBPD, the ICDRC, and the PICS). Nonetheless, none of them are diagnostic tools.

### Sample characteristics

All the participants involved in the studies included in this review were patients with a diagnosis of idiopathic PD. Participants age was around 60–70 years. According to gender information, usually the proportion of males exceeded that of females (i.e., in Papay et al.’s study [55] more than 80% of the sample were males), although other studies included a more gender-balanced sample [51]. Severity of PD was assessed either by the Unified Parkinson’s Disease Rating Scale (UPDRS) [59] or according to the Hoehn and Yahr (HandY) scale [60], though this second scale was the most used. On average, PD patients were at stage 2 according to the HandY scale.

### Qualitative analysis of the psychometric properties

Table 3 displays the assessment of the quality of the psychometric properties that were available for each of the selected instruments according to Terwee et al.’s criteria [44] revised by Park et al. [45].

**Table 3:** Qualitative analysis of the psychometric properties of the selected instruments according to Terwee et al. criteria (2007) revised by Park et al. (2013).

Instrument	Study	Reliability				Validity				Responsiveness	Interpretability
		Internal consistency	Reliability		Measurement error	Content validity	Construct validity		Criterion validity		
			Inter-rater	Test-retest			Structural validity	Hypotheses-testing			
**ASBPD**	Rieu et al. [42]	+	-	-	0	0	+	+	+	0	0
**DDS-PC**	Cabrini et al. [51]	0	0	0	0	+	0	+	0	0	0
**ICDRC**	Baumann-Vogel et al. [53]	0	0	0	0	0	0	+	0	0	0
**PICS**	Okai et al. [54]	0	+	-	0	0	0	+	0	+	0
**QUIP**	Weintraub et al. [49]	0	0	0	0	+	0	+	0	0	0
	Papay et al. [55]	0	-	0	0	0	0	+	0	0	0
	Tanaka et al. [56]	0	0	0	0	0	0	+	0	0	0
	Probst et al. [57]	0	0	0	0	0	0	+	0	0	0
**QUIP-S**	Weintraub et al. [49]	0	0	0	0	0	0	+	0	0	0
	Krieger et al. [58]	0	0	0	0	?	0	0	0	0	0
**QUIP-RS**	Weintraub et al. [50]	0	+	+	0	0	0	+	0	+	0
	Probst et al. [57]	0	0	+	0	?	0	+	0	0	0

*Note*: ASBPD: Ardouin Scale of Behavior in Parkinson’s Disease; DDS-PC: Dopamine Dysregulation Syndrome-Patient and Caregiver Inventory; ICDRC: Impulse Control Disorders and Related Conditions Questionnaire; PICS: Parkinson’s Impulse Control Scale for the severity rating of Impulse-Control Behaviors; QUIP: Questionnaire for Impulsive-Compulsive Disorders in Parkinson’s Disease; QUIP-S: Questionnaire for Impulsive-Compulsive Disorders in Parkinson’s Disease–short version; QUIP-RS: Questionnaire for Impulsive-Compulsive Disorders in Parkinson’s Disease-Rating Scale. + = positive rating, ? = intermediate rating,— = negative rating, 0 = no information available.

#### Reliability

Internal consistency was provided for only one study [42], with moderate values for all the subscales around .70, so, according to Terwee et al.’s criteria [44], the quality of this psychometric property was rated positively.

Examining the second measurement property included in the reliability domain, inter-rater reliability was provided for four studies [42, 50, 54, 55]. However, the entity of different raters is various among the studies; for instance, Papay et al. [55] calculated inter-rater reliability by comparing patient’s answers with an informant’s ones (i.e., a caregiver, such as a close relative); in Weintraub et al.’s study [50], it was computed by comparing the patient’s answers with the assessment of a trained clinician. In the other two studies [42, 54], ratings from different clinicians were compared. According to Terwee et al.’s criteria [44], only Okai et al.’s and Weintraub et al.’s studies were rated positively, since results for the other two studies were lower than the defined criteria. In fact, in Papay et al.’s study kappa values for the agreement between patients and informants for reporting any ICD was .41, and in Rieu et al.’s kappa values ranged from .29 to .81, though only the Hypersexuality behavior met the criterion of kappa being .70 or higher. Referring to test-retest reliability, it was provided for four studies [42, 50, 54, 57]. However, according to Terwee et al.’s criteria, only reliability for Probst et al.’s and for Weintraub et al.’s studies was rated positively, as results met the defined criteria. Instead, weighted kappa was .42 in Okai et al.’s study and ranging in Rieu et al.’s study, from .39 to .79, though only agreement for the Hypersexuality and the Compulsive shopping behaviors reached the minimum criterion of .70.

There was no evidence to evaluate measurement error for the selected instruments.

#### Validity

Concerning validity, it was provided for all studies, though the specific examined aspects of this domain were different among the studies. Referring to face validity, it was provided in few studies [49, 51]. In detail, the QUIP [49] was administered to 10 research members to gather their feedback and to five PD patients and their caregivers. Moreover, regarding the DDS-PC [51], it was reviewed by three patients and caregivers. Still referring to content validity, few studies provided information about the ability to understand the item content. To this extent, concerning the QUIP-S [58], five PD neurologists and 30 patients were asked their comment about the level of comprehension of the questions. Furthermore, eight PD patients and five healthy controls were asked to assess the items readability of the QUIP-RS [57]. However, neither the first nor the second studies reported information about the completeness and/or comprehensiveness of the items, so they were rated as intermediate according to the modifications of Terwee et al.’s criteria [44] proposed by Park et al. [45].

Concerning structural validity, Rieu et al.’ study [42] conducted an Exploratory Factor Analysis (EFA) that forced the number of factors to five, thus explaining the 77% of variance. In detail, factors were 1) Hypodopaminergic disorders, such as depressed mood, 2) Non-motor fluctuations and punding, 3) ICDs, and creativity, 4) Nocturnal hyperactivity, risk-taking behavior, and dopaminergic addiction, and 5) Diurnal somnolence and psychotic symptoms. However, the authors opted for conceptualizing the latter three factors as a unique domain assessing hyperdopaminergic symptoms. Quality of the structural validity for this study was rated positively.

Concerning the hypotheses-testing category, its quality was rated positively for all the studies included in the present review–except for Krieger et al. [58], that did not provide information about this aspect. For instance, the DDS-PC scale showed high correlations for impulsivity measured with the Italian version of the BIS-11 [61], and with the *Cloninger’s Tridimensional Personality Questionnaire* [62], in line with the authors’ hypotheses [51]. In addition, the ASBPD scale [42] showed low association with scales measuring different constructs, such as between each ICD and affective symptoms measured with the *Montgomery and Asberg Depression Rating Scale* [63], and between ICDs and psychotic symptoms assessed with the *Positive and Negative Syndrome Scale* [64].

Hypotheses-testing was also measured in terms of significant differences; in detail, scores at the PICS were significantly different between PD patients with and those without ICDs [54].

Criterion validity was provided for the ASBPD, which showed high correlations with McElroy et al.’s criteria [65] for compulsive shopping, with DSM-IV-TR [5] criteria for pathological gambling, and with Carnes [66] criteria for hypersexuality. Also for this study, quality of the examined psychometric property according to Terwee et al.’s criteria [44] was rated positively.

#### Responsiveness

Responsiveness was tested just for two instruments, namely the QUIP-RS [50], and the PICS [54]. In both cases, the examined tools resulted to show good responsiveness to change after being involved in a clinical treatment for ICDs, and both studies were rated positively according to Terwee et al. criteria [44].

#### Interpretability

There was no evidence to evaluate interpretability for the selected instruments, since mean and SD scores were not presented of at least four relevant subgroups of patients, and MIC were not defined in any of the examined studies.

### Methodological quality of the selected articles

Methodological quality of the investigated measurement properties for the selected studies was judged according to the COSMIN checklist [47, 48]. Results are reported in Table 4. The overall methodological quality was fair to good because of some required information per parameter not being reported; in many cases, methodological quality was rated good instead of excellent because almost all the required data were declared, except for the percentage of missing answers or the procedure of handling missing items [42, 55]. However, there are some exceptions. For instance, both Probst et al.’s study [56] examining the QUIP-RS and Krieger et al.’s study [58] were rated poor when concerning methodological quality of content validity, because of many lacking parameters, since they both examined only the level of comprehension and readability of the questions. Moreover, in Weintraub et al.’s study [50] responsiveness to change was methodologically poor because of the small sample size (i.e., less than 30 patients). On the other hand, in Weintraub et al. study concerning the QUIP [49], methodological quality for content validity was rated excellent, as all the required standards were met. Remarkably, in most cases the generalizability box scored better than the quality of the assessment of the properties per article, as the articles reported most of the required data for this section.

**Table 4:** Methodological quality of reviewed studies according to the COSMIN checklist (Mokkink et al., 2010a, 2010b).

Instrument	Study	Properties assessed	IRT	A	B	C	D	E	F	G	H	I	J	Generalizability
**ASBPD**	Rieu et al. [42]	Internal consistency Reliability Structural validity Hypotheses testing Criterion validity	No	++	++			+	++		++			+++
**DDS-PC**	Cabrini et al. [51]	Content validity Hypotheses testing	No				+		++					++
**ICDRC**	Baumann-Vogel et al. [53]	Hypotheses testing	No						++					++
**PICS**	Okai et al. [54]	Reliability Hypotheses testing Responsiveness	No		0				++			+		++
**QUIP**	Weintraub et al. [49]	Content validity Hypotheses testing	No				+++		++					++
	Papay et al. [55]	Reliability Hypotheses testing	No		++				++					++
	Tanaka et al. [56]	Hypotheses testing	No						++					++
	Probst et al. [57]	Hypotheses testing	No						+					++
**QUIP-S**	Weintraub et al. [49]	Hypotheses testing	No						++					++
	Krieger et al. [58]	Content validity	No				0							++
**QUIP-RS**	Weintraub et al. [50]	Reliability Hypotheses testing Responsiveness	No		+				++			0		++
	Probst et al. [57]	Reliability Content validity Hypotheses testing	No		+		0		+					++

*Note*: ASBPD: Ardouin Scale of Behavior in Parkinson’s Disease; DDS-PC: Dopamine Dysregulation Syndrome-Patient and Caregiver Inventory; ICDRC: Impulse Control Disorders and Related Conditions Questionnaire; PICS: Parkinson’s Impulse Control Scale for the severity rating of Impulse-Control Behaviors; QUIP: Questionnaire for Impulsive-Compulsive Disorders in Parkinson’s Disease; QUIP-S: Questionnaire for Impulsive-Compulsive Disorders in Parkinson’s Disease–short version; QUIP-RS: Questionnaire for Impulsive-Compulsive Disorders in Parkinson’s Disease-Rating Scale. A = internal consistency. B = reliability. C = measurement error. D = content validity. E = structural validity. F = hypothesis testing. G = cross-cultural validity. H = criterion validity. I = responsiveness. J = interpretability. +++ = excellent. ++ = good. + = fair. 0 = poor. Empty boxes = not applicable. IRT = Item Response Theory.

## Discussion

Impulse Control Disorders are one of the most frequent non-motor features of PD, occurring in up to 20% of PD patients [4]. ICDs worsen quality of life and are responsible for several emotional and social impairments [33, 35, 46]. Though not being the only responsible factor for the onset of ICDs, dopaminergic treatment, mainly dopamine agonists, plays an important role [4, 27]. In fact, case reports suggested that ICDs often resolve after adjusting the therapeutic dosage [38, 39]. Thus, to better address patients’ needs and delineate a specific intervention that may include treatment modifications, an accurate assessment of the presence of ICDs is recommended.

As self-report questionnaires and interviews are extremely useful for clinicians to examine ICDs, in the last decade researchers have focused on the development and validation of tools that may support in the clinical assessment. However, despite the implications that ICDs have on PD patients’ lives, these behaviors tend to be under-recognized in clinical practice [33, 37], since routine screening for ICDs is not common [4]. Furthermore, clinicians may often opt for a qualitative, subjective assessment rather than using *ad hoc* designed instruments and protocols, notwithstanding their accuracy, shortness, and ease of use. In addition, not all the existing instruments aiming to assess the same clinical feature are equally good in terms of psychometric properties, since there may be differences in terms of their ability to effectively and precisely measure the considered aspect. As such, before administering a tool, testing its measurement properties would be needed.

To test for the reliability and validity of the existing instruments–so that clinicians may decide to benefit from them–in the present systematic review the quality of the psychometric properties of the included studies was assessed according to Terwee et al.’s criteria [44] revised by Park et al. [45], whereas the methodological quality of the studies was examined according to the COSMIN checklist [47, 48].

Some considerations may be advanced referring to our findings. First, although other instruments specifically assessing only one impulse control disorder in PD patients exist–such as the South Oaks Gambling Screen [67], the Sexual Addiction Screening Test for PD patients (PD-SAST) [68], the Clinician Punding Criteria and Rating Scale for punding [69], and the Saving Inventory-Revised for compulsive hoarding [70]–we decided to include only those instruments that examined more than one ICD at the same time. In fact, to help clinicians in evaluating the presence ICDs in an efficient way, we thought that it would be better to focus on instruments that allowed to assess different ICDs at the same time. Moreover, we included only those instruments whose psychometric properties have been tested. Thus, some screening tools whose psychometric properties have not been examined were not included in the present review.

The second consideration is that further evaluations on the psychometric properties of the instruments examined in this review should be performed, since most studies assessed only two or three psychometric properties. This does not mean that the included measures in this review are not reliable or valid tools. However, as analyses were not exhaustive, further investigation on their psychometric properties is needed. In detail, further information would be needed to provide evidence of internal consistency, measurement error, cross-cultural validity, criterion validity, and responsiveness to change. Additionally, the use of IRT methods for statistical analysis of psychometric properties would also need further exploration. In fact, IRT improves the accuracy of assessment instruments as it examines how well each item can discriminate between people with different levels of the latent trait–in this case, with different severity of ICDs. Furthermore, instead of providing a single value for reliability (e.g., alpha coefficient), through the test information function (TIF) IRT allows for assessment of measurement precision at different levels of the measured construct [71], meaning that the more information the test provides at a particular trait level, the smaller the error associated with estimation is and the higher reliability is.

Concerns may also arise about the quality of the examined psychometric properties, as, in some studies, results were not good enough to be rated positively according to Terwee et al.’s criteria [44]. Furthermore, in some cases methodological quality for some measurement properties was doubtful or lacking, so future studies should re-perform the analyses with a more methodologically precise procedure. For instance, studies should better focus on and report the preliminary data analyses referring to the percentage of missing answers and their handling procedure.

In sum, from an overall view that considers the quality of the measurement properties of the instruments, the methodological quality of their related article, and the number of articles that focused their analyses on a specific tool, the QUIP and the ASBPD seem to be better from a psychometric point of view. The QUIP may be selected when looking for a quick tool aiming to explicitly assess ICDs, whereas the ASBPD may be chosen when aiming to gather more information about the general psychological status of PD patients. However, these two measures–above all, the QUIP–represent the most studied, so the most available information is about them. Thus, future studies should further examine the psychometric properties of the other tools. From a clinical point of view, while the ASBPD and the ICDRC are behavioral scales that only assess the frequency and intensity of symptoms–though the ICDRC also examines their consequences–the other instruments investigate many dimensions underlying ICDs–such as thinking too much about those behaviors, having urges or desires to engage in them, and experiencing difficulty controlling them. Thus, those measures result to be more informative on which factors may contribute to ICDs, allowing to obtain a more detailed frame of the patient and his behaviors.

Despite the contribution, this review may present some limitations concerning which tools should have been included. Though EMBASE, MEDLINE, and PsycINFO index thousands of journals, some studies examining the psychometric properties of ICDs instruments in PD patients may have not been included because they were published in journals not indexed in the three examined databases. Moreover, the exclusion criterion of being meeting abstracts or materials published in the form of thesis, book chapters, and manuals may have led to the omission of relevant results.

Although the gold standard for diagnosis still remains a detailed diagnostic interview, we hope that this review will encourage clinicians to get acquainted with the use of valid and reliable tools in their everyday clinical practice, rather than relying on an incomplete evaluation to assess ICDs in PD patients. Furthermore, this review will be helpful in the process of instrument selection by allowing researchers and clinicians to easily evaluate and choose the most appropriate measures that fulfil their purpose.

## Supporting information

S1 Table PRISMA 2009 checklist. (DOCX)
