# Supplementary material for: Impulse control disorders in Parkinson’s disease: A systematic review on the psychometric properties of the existing measures
Source: PLoS One. 2019 Jun 4;14(6):e0217700. doi: 10.1371/journal.pone.0217700 (PMC6548365; doi:10.1371/journal.pone.0217700)
Supplement: S1 Table — (DOCX) [file pone.0217700.s001.docx]

**Faculty of Engineering and Physical Sciences**

**Ethics Application**

**Screening Questionnaire**

|  |  | **Yes** | **No** |
| --- | --- | --- | --- |
|  | Do you have ethical approval for the study from another recognised Ethics Committee?  *If* ***yes,*** *you should complete the form, Confirmation of Existing Ethical Approval.* |  |  |
|  | Are the proposed participants under the jurisdiction of the prison service?  *If* ***yes****, you will need to apply to the relevant prison ethics committee to obtain ethical approval. You will also need to apply for Faculty REC Approval by completing the Full Ethical Approval Form.* |  |  |
|  | Are the proposed participants to be recruited from NHS organisations, including hospitals, social care homes, residential care homes, nursing homes?  *If* ***yes****, you will need to apply to the relevant NHS Research Ethics Committee to obtain ethical approval*. |  |  |
|  | Does this study involve animals?  *If* ***yes*** *and the procedures are covered by the Animal (Scientific Procedures) Act 1986, an application should be submitted to AWERB*  *If* ***yes*** *and the procedures are not covered by the Animal (Scientific Procedures) Act 1986, please complete the form, Animal Welfare Ethical Approval Form. This should be requested by emailing the Research Ethics Officer, at* [*facultyreceps@qub.ac.uk*](mailto:facultyreceps@qub.ac.uk) |  |  |

**Prior to completion of this application please ensure that you have**

*i. Finalised the protocol*

*ii. Undertaken peer review in accordance with School and University procedures*

*iii. Completed any relevant Health and Safety, and/or Risk Assessment procedures*

**Consideration for Proportionate Review**

Please check the correct box for each question and read the criteria at the end of the screening tool to determine if your application may be considered by the Proportionate Review system or the Full Ethical Approval system.

|  |  | **Yes** | **No** |
| --- | --- | --- | --- |
|  | Is it your intention to conduct research involving children under 16 with no physical, mental or learning impairment?  *You may submit an application under the Proportionate Review system if this is ‘Yes’. You must indicate in the “Dealing with ethical issues box” how consent will be obtained from the participant’s parents / guardians or responsible adult and how assent will be obtained from the participants.* |  |  |
|  | Is it your intention to conduct research involving potentially vulnerable groups, individuals with mental or learning or behavioural disabilities, victims of crime or events which are stress inducing (e.g. terrorist attacks)? |  |  |
|  | Does your research involve access to records of a personal nature or confidential information (including genetic, health or other biological information) for which specific consent has not been granted for its use for the purposes of the research and it is not anonymised? |  |  |
|  | Does your research involve access to potentially sensitive data through third parties (such as employee data)? |  |  |
|  | Does your research involve a questionnaire which seeks responses about highly sensitive topics for example, sexual behaviour, illegal behaviour, political opinion, religious or spiritual beliefs, race or ethnicity, experience of violence, abuse or exploitation, mental health where the individual’s response can be identified and attributed to the individual? |  |  |
|  | Does your research involve a 1:1 interview with participants which seeks responses about highly sensitive topics for example, sexual behaviour, illegal behaviour, political opinion, religious or spiritual beliefs, race or ethnicity, experience of violence, abuse or exploitation, mental health? |  |  |
|  | Does your research involve a group ‘discussion’ forum which seeks responses about highly sensitive topics for example, sexual behaviour, illegal behaviour, political opinion, religious or spiritual beliefs, race or ethnicity, experience of violence, abuse or exploitation, mental health? |  |  |
|  | Does your research involve respondents participating through social media about highly sensitive issues where the individual’s response can be identified and attributed to the individual? |  |  |
|  | Does your research involve the collection of human tissue of biological samples from the individual, e.g. saliva? |  |  |
|  | Does your research involve actions with potentially adverse environmental impact? |  |  |
|  | Does your research involve a significant element of deception? |  |  |
|  | Does your research involve invasive procedures, e.g. the administration of drugs or other substances (e.g. food, supplements), vigorous physical exercise, or techniques such as hypnotherapy that would not usually be encountered in everyday life? |  |  |
|  | Does your research involve visual/vocal methods where participants or other individuals may be identifiable in the images used or generated, where consent for the use of the images in research has not been obtained? |  |  |
|  | Does your research involve financial inducements (other than reasonable expenses and compensation for time) being offered to participants? |  |  |
|  | Does your research involve covert observation of individuals in non-public places without their consent? |  |  |
|  | Could participation in the research result in psychological stress, anxiety or humiliation or more than minimal pain, that that would not usually be encountered in everyday life? |  |  |
|  | Could participation in the research result in repetitive or prolonged testing such that it may induce fatigue or adverse physical or psychological states? |  |  |
|  | Could participation in the research result in behavioural change through therapeutic interventions, for example, the administration of substances or enrolment in programmes with the specific aim of altering behaviour? |  |  |
|  | Could participation in the research result in ‘labelling’ either by the researcher (e.g. categorisation) or by the participant (e.g. ‘I am stupid’, ‘I am not normal’)? |  |  |
|  | Could participation in the research result in an adverse impact on employment or social standing (e.g. discussion of an employer, discussion of commercially sensitive information)? |  |  |
|  | Could participation in the research result in findings relevant for an individual participant’s health and well-being? |  |  |

If all the answers are **no** then you may complete the Proportionate Review Ethics Application.

If the answer to question 1 is **yes** you may still complete the Proportionate Review Ethics Application but must provide the information required as specified above in the application.

If answers to the questions are yes, other than 1, you must complete the Full Ethics Application.

Please indicate the type of project:

Staff:  Undergraduate (UG):  Postgraduate Taught (PGT):

Postgraduate Research (PGR):

Chief Investigator (This must be a member of QUB staff. For student projects, the Supervisor should be named as the Chief Investigator.)

| Name | Viola Angela Izzo |
| --- | --- |
| Staff Number | 2624816 |
| School | Psychology |
| Email Address | violaangela.izzo@unifi.it |

QUB Co-investigators or Students involved

| Name | Staff/Student number | School | Email Address |
| --- | --- | --- | --- |
| Kinga Morsanyi | 3047759 | Psychology | k.morsanyi@qub.ac.uk |
| Click here to enter text. | Click here to enter text. | Click here to enter text. | Click here to enter text. |
| Click here to enter text. | Click here to enter text. | Click here to enter text. | Click here to enter text. |
| Click here to enter text. | Click here to enter text. | Click here to enter text. | Click here to enter text. |
| Click here to enter text. | Click here to enter text. | Click here to enter text. | Click here to enter text. |
| Click here to enter text. | Click here to enter text. | Click here to enter text. | Click here to enter text. |

Non-QUB co-investigators

| Name | Institution/Company | Email Address |
| --- | --- | --- |
| Caterina Primi | University of Florence | caterina.primi@unifi.it |
| Click here to enter text. | Click here to enter text. | Click here to enter text. |
| Click here to enter text. | Click here to enter text. | Click here to enter text. |
| Click here to enter text. | Click here to enter text. | Click here to enter text. |
| Click here to enter text. | Click here to enter text. | Click here to enter text. |
| Click here to enter text. | Click here to enter text. | Click here to enter text. |
| Click here to enter text. | Click here to enter text. | Click here to enter text. |

| Full Title of Research | **Exploring the relation between Math anxiety and Math performance using skin conductance measures.** |
| --- | --- |
| Abbreviated Running Title | Math anxiety and math performance |
| Proposed Start Date | 07/01/2019 |
| End Date | 31/12/2019 |

**Faculty of Engineering and Physical Sciences**

**Full Ethics Application**

|  | Outline briefly the aims and rationale of your study. Include the main research questions(s) |
| --- | --- |
| The Yerkes-Dodson Law states that there exists a curvilinear relation between anxiety and performance in a given task, according to which moderate levels of anxiety are more beneficial to performance than both very low and very high levels of anxiety. Despite previous studies have found practical confirms of this law when examining general anxiety, a curvilinear relation between mathematics anxiety (i.e., anxious state in response to mathematics-related situations) and mathematics performance has not been found. In fact, results showed the existence of a negative linear relation, thus not supporting the existence of an optimal, moderate level of math anxiety. However, previous studies usually examined math anxiety using self-reported measures, which are susceptible to response bias, rather than using physiological measures such as skin conductance. Moreover, other cognitive and attitude variables, including working memory or academic/mathematics motivation, have found to mediate or moderate the relation between mathematics anxiety and performance. Thus, the non-confirmation of the principles of the Yerkes-Dodson Law when examining maths anxiety and performance may be due to those reasons. With these premises, the present study aims at examining the nature of the relationship between maths anxiety and math performance not only using both self-reported and physiological measures such as skin conductance, but also taking into consideration several possible mediator or moderator variables. In details, the present study aims at examining the following questions: 1. Does mathematics anxiety display a linear negative relationship with performance in an arithmetic task, or does it conform to the Yerkes-Dodson Law? 2. Does the relationship between skin conductance/mathematics anxiety and performance in an arithmetic task differ as a result of task difficulty and time pressure during the task?  3. How do several cognitive and attitude variables, including working memory capacity, test anxiety, subjective numeracy, cognitive reflection, and academic motivation influence the relation between mathematics anxiety (measured with skin conductance) and mathematics performance? | |

|  | Outline briefly the methods and analysis you intend to use. |
| --- | --- |
| The study will test the relationship between performance on an arithmetic task and mathematics anxiety, measured both using a self-report measure and skin conductance over the course of the arithmetic task (measured in microsiemens - µS). Performance on the arithmetic task will be quantified based on the number of questions the participant answered correctly. To measure skin conductance during the task assessing math performance, the Biopac base module (MP150), an electrodermal activity amplifier (Biopac GSR100C), two reusable electrodes and a computer running the ‘AcqKnowledge’ software. Both the amplifier and electrodes operate using low voltage batteries. The electrodes will be treated with an electrolyte gel prior to testing, and will be attached to the participants’ index and middle fingers using Velcro strips and special gloves.  The study will also test whether this relationship differs as a result of the general difficulty of the questions and whether the questions were administered under time pressure. Moreover, the study will test how the relationship between mathematics anxiety and performance may be either mediated or moderate by several cognitive and attitude variables, including working memory capacity, test anxiety, subjective numeracy, cognitive reflection, and academic motivation. All those variables will be assessed using self-reported measures. Pearson’s r correlations, regression analyses, as well as mediation and moderation analyses will be performed. | |

**Potential Ethical Issues**

|  | Does the study require participants to disclose information of a sensitive or personal nature?  *If yes, say why this is necessary and what steps have been taken to minimise any adverse effects* | **Yes** | **No** |
| --- | --- | --- | --- |
| N/A | | | |

|  | Does the research have the potential to cause adverse environmental impact?  *If yes, say why this is necessary and what steps have been taken to minimise any adverse effects* | | | **Yes** | | **No** | |
| --- | --- | --- | --- | --- | --- | --- | --- |
| N/A | | | | | | | |
|  | | Does the study involve any significant deception or withholding information?  *If yes, say why this is necessary and what steps have been taken to minimise any adverse effects* | **Yes** | | **No** | |  |
| N/A | | | | | | |  |

|  | Does the study involve invasive procedures, e.g. the administration of drugs or other substances (e.g. food, supplements), vigorous physical exercise, or techniques such as hypnotherapy that would not usually be encountered in everyday life?  *If yes, say why this is necessary and what steps have been taken to minimise any adverse effects* | **Yes** | **No** |
| --- | --- | --- | --- |
| The study involves the measurement of skin conductance, which is not usually encountered in everyday life. There is a small risk that the participants will experience skin irritation from the electrodermal gel applied to the electrodes. However, the gel that will be used is hypoallergenic, so this event is unlikely to occur. | | | |

|  | Does the study involve any visual/vocal methods where participants or other individuals may be identifiable in the images used or generated, where consent for the use of the images in research has not been obtained?  *If yes, say why this is necessary and what steps have been taken to minimise any adverse effects* | **Yes** | **No** |
| --- | --- | --- | --- |
| N/A | | | |

|  | Does the study involve the covert observation of individuals in non-public places without their consent?  *If yes, say why this is necessary and what steps have been taken to minimise any adverse effects* | **Yes** | **No** |
| --- | --- | --- | --- |
| N/A | | | |

|  | Does the study involve any psychological risk (e.g. stress, anxiety or humiliation) that would not usually be encountered in everyday life?  *If yes, say why this is necessary and what steps have been taken to minimise any adverse effects* | **Yes** | **No** |
| --- | --- | --- | --- |
| If the participants struggle with mathematics, they may experience some stress when carrying out the harder arithmetic problems under time pressure. However, participants will be told when they are about to start a set of hard questions or whether the questions are given under time pressure, which should reduce the possibility of significant stress being caused through shock. In addition to this, the participants will be ensured that their data are anonymous, so they should feel less stressed knowing that no-one other than the researcher will able to see their results. They will also be assured that they will be free to withdraw from the study at any time. | | | |

|  | Does the study involve any physical risk (e.g. more than minimal pain, fatigue) that would not usually be encountered in everyday life?  *If yes, say why this is necessary and what steps have been taken to minimise any adverse effects* | **Yes** | **No** |
| --- | --- | --- | --- |
| There is a small chance that the participant will experience skin irritation from the electrodermal gel applied to the electrodes. However, the gel that will be used is hypoallergenic, so this is unlikely. | | | |

|  | Does the study intentionally aim to result in a change in behaviour in the participant?  *If yes, say why this is necessary and what steps have been taken to minimise any adverse effects* | **Yes** | **No** |
| --- | --- | --- | --- |
| N/A | | | |

**Participants**

|  |  | **Experimental** | **Controls** |
| --- | --- | --- | --- |
|  | How many participants will be involved in the study | About 20 | N/A |
|  | How many will be students at QUB | About 20 | N/A |
|  | How many will be adults outside QUB | 0 | N/A |
|  | How many will be individuals aged 15 and under | 0 | N/A |
|  | How many will be individuals with specific medical conditions | 0 | N/A |

|  | How will participants be recruited? |
| --- | --- |
| The participants will be recruited through SONA, email and word of mouth. | |

|  | What, if any, inclusion or exclusion criteria will be used? |
| --- | --- |
| The sample will exclude students from inherently mathematical courses (Mathematics, Accounting, Physics, Chemistry, Engineering, Computer Science, Economics etc.) | |

|  | What, if any, is the relationship between the investigators and participants (e.g. fellow students, club members, family friends)? |
| --- | --- |
| No relationships exist between the investigators and participants. | |

|  | How and what will individuals be told about the research?  *(a copy of the Participant Information Sheet must be attached to this application)* |
| --- | --- |
| The participant will be given an information sheet which briefly explains the purpose of the investigation. The participant will be informed of each questionnaire and task they have to complete. | |

|  | How will participants provide consent?  *(a copy of the Consent Form (if applicable) must be attached to this application)* |
| --- | --- |
| The School of Psychology standard consent form will be used for paper and pencil data collection. | |

|  | If individuals are unable to give consent, e.g. through age or incapacity, how will consent be obtained? |
| --- | --- |
| The participants who are unable to give consent will not be recruited. | |

|  | Can the participants withdraw from the research at any time?  *How and when are individuals informed of this?* | **Yes** | **No** |
| --- | --- | --- | --- |
| Participants will be informed that they can withdraw from the study at any time during the study, and up to 2 weeks after participation. This information will be included both in the recruitment materials and in the Consent Form. | | | |

|  | If individuals wish to withdraw, what will happen to them and their data? |
| --- | --- |
| If they withdraw, their data will be deleted. There will be no other consequences of withdrawal. The participants who decide to withdraw during the lab-based study will be thanked for their interest in the study, and they will be offered an opportunity to ask any questions that they might have. | |

|  | Are participants being offered any financial inducements (other than reasonable expenses and compensation for time) to participate?  *If yes, explain why this is necessary* | **Yes** | **No** |
| --- | --- | --- | --- |
| N/A | | | |

|  | Does the research involve access to records of a personal nature or confidential information (including genetic, health or other biological information) for which specific consent has not been granted for its use for the purposes of the research and it is not anonymised or potentially sensitive data through third parties (such as employee data)?  *If yes, explain how the confidentiality of the information will be preserved* | **Yes** | **No** |
| --- | --- | --- | --- |
| N/A | | | |

|  | Could the study result in ‘labelling’ either by the researcher (e.g. categorisation) or by the participant (e.g. ‘I am stupid’, ‘I am not normal’), or an adverse impact on employment or social standing (e.g. discussion of an employer, discussion of commercially sensitive information)?  *If yes, explain how the confidentiality of the information will be preserved* | **Yes** | **No** |
| --- | --- | --- | --- |
| N/A | | | |

|  | Could the study reveal findings relevant for an individual participant’s health and well-being?  *If yes, please state what information the individual will be given, what permission will be obtained, and describe how the information will be handled, e.g. who will it be passed on to.* | **Yes** | **No** |
| --- | --- | --- | --- |
| N/A | | | |

**Methods**

|  | Will you be administering any substances to participants? | **Yes** | **No** |
| --- | --- | --- | --- |
|  | Will you be asking participants to refrain from taking any substance they would usually take? |  |  |
| N/A | | | |

|  | Will you be administering any questionnaires to participants? | **Yes** | **No** |
| --- | --- | --- | --- |
|  | Will you be undertaking any interviews or semi-structured interviews? |  |  |
| 1a. Abbreviated Math Anxiety Scale 1b. Hopko, D. R., Mahadevan, R., Bare, R. L., & Hunt, M. K. (2003). The abbreviated math anxiety scale (AMAS) construction, validity, and reliability. Assessment, 10(2), 178-182. 1c. To measure the participant's level of mathematics anxiety. 2a. Test Anxiety Inventory 2b. Spielberger, C. D. (2010). Test anxiety inventory. John Wiley & Sons, Inc. 2c. To measure the participant's level of test anxiety. 3a. Subjective Numeracy Scale 3b. Fagerlin, A., Zikmund-Fisher, B. J., Ubel, P. A., Jankovic, A., Derry, H. A., & Smith, D. M. (2007). Measuring numeracy without a math test: development of the Subjective Numeracy Scale. Medical Decision Making, 27(5), 672-680. 3c. To measure the participant's level of subjective numeracy. 4a. Cognitive Reflection Test  4b. Frederick, S. (2005). Cognitive reflection and decision making. Journal of Economic perspectives, 19(4), 25-42. 4c. It is a measure of reflection impuslvity. 5a. Academic Motivation Scale. 5b. Vallerand, R. J., Pelletier, L. G., Blais, M. R., Briere, N. M., Senecal, C., & Vallieres, E. F. (1992). The Academic Motivation Scale: A measure of intrinsic, extrinsic, and amotivation in education. Educational and psychological measurement, 52(4), 1003-1017. 5c. To measure the participant's academic motivation.  Examples of items for each questionnaire are provided in the Materials document. | | | |

|  | Is permission required from any other source before commencing the research or for the use of equipment?  *If yes, state what permission is required and provide evidence* | **Yes** | **No** |
| --- | --- | --- | --- |
| N/A | | | |

**Data security and participant confidentiality**

|  | Will data be anonymised such that individual responses cannot be identified?  *If yes, describe how you will do this* | **Yes** | **No** |
| --- | --- | --- | --- |
| Participant numbers will be used to link the results from different tasks together. Consent forms will record the participant numbers in case the participants want to withdraw their data after participation. Consent forms will be stored separately from response sheets. | | | |

|  | If data is not anonymised describe what steps will be taken to preserve the confidentiality of the data. |
| --- | --- |
| N/A | |

|  | Where will all forms of the data be stored? |
| --- | --- |
| Data will be collected in both paper-and-pencil and electronic format, but eventually all data will be stored electronically. Once paper and pencil data has been recorded electronically, the original response sheets will be destroyed, and data will only be stored electronically on password protected computers. Only the investigators will have access to the data. In case laptops are used for data storage, the laptops will be encrypted. | |

|  | Who will have access to the data? |
| --- | --- |
| The named investigators. | |

|  | Where will Consent Forms be stored? |
| --- | --- |
| The consent forms will be stored in the investigator’s office in a locked filing cabinet. | |

|  | Will individually identifiable information be given to third parties or available through publications, etc?  *If yes, state why this is necessary and demonstrate that participants are made aware of this.* | **Yes** | **No** |
| --- | --- | --- | --- |
| N/A | | | |

**To be confirmed by Chief Investigator / Supervisor:**

| I confirm that the protocol has been peer reviewed in accordance with School and University procedures and is deemed to be viable and scientifically valid |  |
| --- | --- |
| I confirm that the relevant risk assessment and health and safety protocols in relation to this research have been undertaken and appropriate safeguards in place to manage any risks. |  |
| I believe that the research does not raise significant ethical issues. |  |
|  |  |
| **By submitting this application all applicants confirm:** |  |
| I will preserve the confidentiality of all information provided by participants in this research. |  |
| I will abide by the procedures established by the University, relevant professional bodies and other organisations in conducting this research. |  |
| I will conduct the research in accordance with the protocol supplied. |  |
| ****For applications from the School of Psychology:***  I have consulted the BPS Code of Human Research Ethics. |  |
| ****For applications from all other Schools:*** I have consulted the appropriate Codes of Practice for my professional body. |  |

Signature of Chief Investigator/Supervisor:

(Electronic signature acceptable)
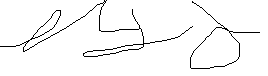


QUB Staff Number of Chief Investigator/Supervisor: 3047759

Date of Submission:

This application form and the material indicated below should be submitted by email to:

[facultyreceps@qub.ac.uk](mailto:facultyreceps@qub.ac.uk)

Forms to be submitted (Please ensure all submissions begin with the SURNAME of the Chief Investigator or Supervisor):

- Full Ethical Approval form
- Research Protocol
- Participant Information Sheet(s)
- Participant Consent Form(s)
- Any relevant permissions, e.g. to access participants
- Any unpublished questionnaires or interview schedules
- Any other information (list below)
- The Materials Form

Please ensure that all study documents are marked with a version number and date.

If you require any information in respect of the above application, please contact the University Research Ethics Officer, [facultyreceps@qub.ac.uk](mailto:facultyreceps@qub.ac.uk), tel 2529.
